# Monitoring wetland water quality related to livestock grazing in amphibian habitats

**DOI:** 10.1007/s10661-020-08838-6

**Published:** 2021-01-13

**Authors:** Kelly L. Smalling, Jennifer C. Rowe, Christopher A. Pearl, Luke R. Iwanowicz, Carrie E. Givens, Chauncey W. Anderson, Brome McCreary, Michael J. Adams

**Affiliations:** 1 U.S. Geological Survey, New Jersey Water Science Center, Lawrenceville, NJ 08648 USA; 2 U.S. Geological Survey, Forest and Rangeland Ecosystem Science Center, Corvallis, OR 97331 USA; 3 U.S. Geological Survey, Leetown Science Center, Kearneysville, WV 25430 USA; 4 U.S. Geological Survey, Upper Midwest Water Science Center, Lansing, MI 48911 USA; 5 U.S. Geological Survey, Oregon Water Science Center, Portland, OR 97201 USA

**Keywords:** Amphibian, Estrogenicity, Fecal indicator bacteria (FIB), Monitoring, Nutrients, *Rana pretiosa*, Wetland

## Abstract

Land use alteration such as livestock grazing can affect water quality in habitats of at-risk wildlife species. Data from managed wetlands are needed to understand levels of exposure for aquatic life stages and monitor grazing-related changes afield. We quantified spatial and temporal variation in water quality in wetlands occupied by threatened Oregon spotted frog (*Rana pretiosa*) at Klamath Marsh National Wildlife Refuge in Oregon, United States (US). We used analyses for censored data to evaluate the importance of habitat type and grazing history in predicting concentrations of nutrients, turbidity, fecal indicator bacteria (FIB; total coliforms, *Escherichia coli* (*E. coli*), and enterococci), and estrogenicity, an indicator of estrogenic activity. Nutrients (orthophosphate and ammonia) and enterococci varied over time and space, while *E. coli*, total coliforms, turbidity, and estrogenicity were more strongly associated with local livestock grazing metrics. Turbidity was correlated with several grazing-related constituents and may be particularly useful for monitoring water quality in landscapes with livestock use. Concentrations of orthophosphate and estrogenicity were elevated at several sites relative to published health benchmarks, and their potential effects on *Rana pretiosa* warrant further investigation. Our data provided an initial assessment of potential exposure of amphibians to grazing-related constituents in western US wetlands. Increased monitoring of surface water quality and amphibian population status in combination with controlled laboratory toxicity studies could help inform future research and targeted management strategies for wetlands with both grazing and amphibians of conservation concern.

**Supplementary Information:**

The online version contains supplementary material available at 10.1007/s10661-020-08838-6.

## Introduction

Livestock grazing is one of the most common land uses globally (Bigelow and Borchers 2012) and in the western United States (US), grazing is widespread across a broad range of landscapes and wildlife habitats (Fleischner 1994). Grazing can alter the biotic, chemical, and physical properties of habitats, but many grazed areas also support high biodiversity (Milchunas et al. 1998; Verga et al. 2012). Effects of grazing tend to vary by watershed hydrology and geology, site-specific grazing histories, climate, changes in land use, and current best management practices (Scrimgeour and Kendall 2002). Species relying on aquatic habitats in grazed landscapes, such as amphibians, show diverse responses to grazing pressures (Howell et al. 2019).

Amphibians are experiencing worldwide declines, with nearly 1 in 3 species at risk of extinction (Stuart et al. 2004). Habitat loss and alteration are primary drivers of declines (Collins and Storfer 2003), yet in areas where water is scarce, stock ponds, wetlands, and small streams used by cattle can provide essential aquatic habitat (Knutson et al. 2004). Water quality changes associated with grazing (i.e., increased nitrate or sediment loads, reduced dissolved oxygen, introduction of steroid hormones) have potential to alter amphibian abundance, growth, species richness, sex ratios, behavior, and parasite communities (Marco et al. 1999; Johnson and Chase 2004; Kolodziej and Sedlak 2007; Burton et al. 2009; Lambert et al. 2015; Babini et al. 2016). However, in some systems, the addition of nutrients can benefit amphibians via increased habitat productivity and resource allocation (Plăiaşu et al. 2010).

The Oregon spotted frog (*Rana pretiosa*; *R. pretiosa*) is a threatened amphibian inhabiting emergent wetlands in the Pacific Northwest where grazing is a common land use (USFWS 2014). Grazing has been used as a management strategy to control invasive vegetation and enhance open water breeding habitat for *R. pretiosa* (Watson et al. 2003). All stages (egg, larva, juvenile, subadult, and adult) of *R. pretiosa* are strongly aquatic and thus directly tied to water chemistry. However, within habitats used by *R. pretiosa*, water quality changes associated with grazing or other land uses are largely undescribed. Field data collected from other sites in the western US have noted inconsistent effects of grazing on water quality and biotic responses in amphibian habitats dependent on stocking density, habitat type, and measured water quality constituents. For example, at a *Rana luteiventris* site in northeastern Oregon, Adams et al. (2009) detected no significant differences in water quality, including measures of nutrients and specific conductance, before and after grazing exclosure treatments. Similarly, Roche et al. (2012) found no significant cattle fencing treatment effect on water quality at Sierra Nevada wet meadow *Anaxyrus canorus* sites in California. Other studies have identified links between grazing and nitrogenous compounds (e.g., Joseph et al. 2016), fecal bacteria (e.g., Gary et al. 1983), and turbidity (e.g., Campbell and Allen-Diaz 1997). The inconsistent effects of cattle grazing on water quality in amphibian habitats emphasize the need for field studies prioritizing species of conservation concern such as *R. pretiosa* in order to build context in which monitoring can inform expectations and adjustments in land management.

Grazing is commonly employed on public lands within the National Wildlife Refuge System mandated to conserve wildlife such as amphibians. Grazing and habitat improvement can be compatible goals, but resource managers require data on measurable indicators of grazing to gauge the success of current practices and inform management alternatives. We assessed water quality in *R. pretiosa* habitat at Klamath Marsh National Wildlife Refuge (KMNWR) in the upper Klamath Basin of south-central Oregon, US. Our primary objective was to collect baseline water quality data to characterize conditions to which *R. pretiosa* on the refuge are potentially exposed. We were particularly interested in identifying relationships among several potentially important water quality constituents and determining how well habitat and grazing conditions potentially predict exposure. These data were requested by public land managers in the region to better understand how to monitor water quality and habitat conditions where grazing is a prominent land use.

## Materials and methods

### Description of study system

The KMNWR encompasses 16,546 ha (ha) of wetland and upland habitats along the Williamson River. About 12,000 ha of the refuge is wetland habitat comprising emergent, shrub, palustrine, and lacustrine types (USFWS 2010). Water levels are highest in the spring and early summer and influenced by the Williamson River, Big Springs, other small seasonal tributaries, regional ground-water discharges, and snow melt. Ditches on KMNWR were built to drain marshes and deliver water to lands on and off the refuge.

**Fig. 1:**
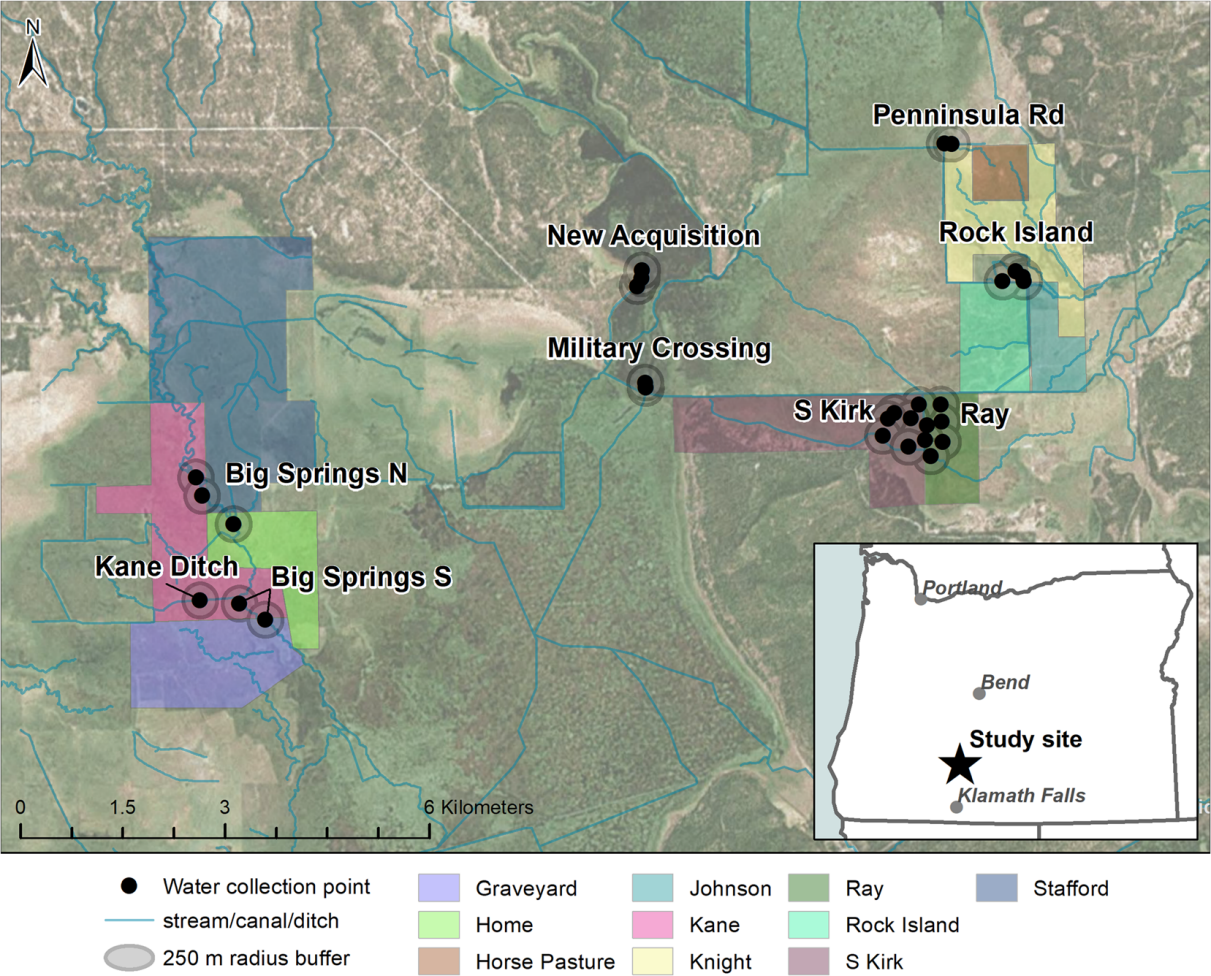
Map of study area in Klamath Marsh National Wildlife Refuge in Oregon. Water collection *points* are given as 1–6 black dots (at mean center of samples collected 2017–2018) within labeled sites. Gray circles around points represent a 250-m radius used to quantify grazing that might influence each sample. Grazing units (pastures) are represented by shaded colored areas

We collected water samples from habitats used by *R. pretiosa* at KMNWR (Fig. 1). Water quality constituents are of direct relevance to understanding potential exposure of *R. pretiosa* because all life stages are aquatic (Pearl and Hayes 2005). Eggs are laid in spring (usually April in KMNWR) in sunny shallows, and developing larvae use benthic detritus in a range of depths (Pearl and Hayes 2004). Larvae transform in the late summer and juveniles use aquatic and riparian habitats for dispersal and local migration among seasonal habitats. Juveniles and adults bask and forage in deeper pools in summer, and usually winter submerged in waters that do not freeze.

In consultation with refuge staff, we assembled records for each grazing unit surrounding our sampling locations (Fig. 1; Table S1). Information on the number and timing of cattle on each pasture was available for 2010–2018, prior to which records were less detailed and grazing tended to be sporadic and of low intensity. Grazing was mostly confined within boundaries of refuge administrative units and neighboring private pastures. When trespass was noted, total acreage grazed was adjusted to account for the trespassed area. We used Esri ArcMap (version 10.6.1) to estimate the size of the area grazed in each pasture (ESRI 2018).

### Selection of water quality constituents, sample collection, and analysis

We focused our water quality assessment on constituents that have been used previously in other grazing studies and have the potential to adversely affect *R. pretiosa* and other amphibian species including nutrients, turbidity, fecal indicator bacteria (FIB; total coliforms, *Escherichia coli* (*E. coli*), and enterococci), and estrogenicity. We collected surface water samples in June, August, and September in 2017, and June, July, August/September, and October of 2018 (Rowe et al. 2019) using standard methods (U.S. Geological Survey 2006). Sample collection coincided with grazing intensity on the refuge and regional climatic conditions (e.g., cool, wet winters and warm, dry summers) and wetland hydrologic conditions (e.g., maximum water depth in the spring/early summer from residual snow melt). We collected 1–6 water samples (scaled to site size) distributed approximately evenly around each site during each sampling event and placed on ice in the field (Table S1). Prior to sample collection, we measured water temperature, pH, and specific conductance with a YSI ProDSS multiparameter meter. We visually determined whether there was directional flow at the sampling location; locations with a current were categorized as *lotic = yes*, and lentic habitats with no flow were categorized as *lotic = no*. At a random subsample of water sampling points, we also collected field replicates and field blanks consisting of sterile deionized water. We analyzed field samples, field replicates, and field blanks for each constituent. Field blanks did not contain detectable levels of the analyzed constituents, and field replicates were within acceptable ranges for each constituent based on laboratory quality assurance/quality control standards. Water samples were shipped on ice to the respective laboratories and stored at 4 °C prior to analysis within their specified holding times.

Water samples for dissolved nutrients were collected in 1-L polyethylene bottles, field filtered (0.45 μm), and analyzed for ammonia as nitrogen (ammonia), nitrite as nitrogen (nitrite), nitrate plus nitrite as nitrogen (nitrate + nitrite), and orthophosphate as phosphorous (orthophosphate) using colorimetric determination (Fishman 1993; Patton and Kryskalla 2011). Detection limits (DL) for ammonia, nitrite, nitrate + nitrite, and orthophosphate were 0.01 mg/L, 0.01 mg/L, 0.001 mg/L, and 0.004 mg/L, respectively. For suspended particulates (turbidity), unfiltered water was collected in a 500-mL clear polyethylene bottle and analyzed using Hach 2100AN (USEPA 1993); the DL was 2 NTRU (nephelometric turbidity ratio unit; Anderson 2005).

Water samples for FIB were collected in 500-mL polyethylene bottles and analyzed for total coliforms, *E. coli*, and enterococci using the IDEXX (Westbrook, Maine), 24-h Colilert (American Public Health Association 1998; American Public Health Association 2017), and IDEXX Enterolert (American Public Health Association 2017) most probable number method, respectively. The DL for total coliforms, *E. coli*, and enterococci was < 1 MPN/100 mL for undiluted samples. For diluted samples, the DL was adjusted to < 10 MPN/100 mL. Total coliforms and enterococci also included values above the linear range of a standard curve (i.e., levels exceeded the assay’s quantification limits).

Estrogenicity was measured as the net activation of estrogenic compounds in water sample extracts relative to 17β-estradiol (E2) using the bioluminescent yeast estrogen screen (BLYES) (Sanseverino et al. 2005; Ciparis et al. 2012). Estrogenicity has been broadly used as an indicator of natural and anthropogenic estrogens in surface water (Ciparis et al. 2012) and was included in the current study to assess the potential exposure of *R. pretiosa* to endogenous steroidal estrogens (naturally occurring female hormones) excreted from livestock (Hanselman et al. 2003; Kolodziej and Sedlak 2007). Exposure to estrogens has the potential to act directly on the endocrine system resulting in intersex and immunosuppression (Lambert et al. 2015; Lambert and Skelly 2016) in certain amphibian species. Water samples for estrogenicity were collected in 1-L amber bottles, acidified, and filtered prior to extraction. The DL for estrogenicity was 0.23 ng/L.

### Statistical analysis

#### Explanatory variables

We developed three spatially nested grouping levels for our water samples: *point*, *site*, and *cluster* (Table 1). We defined *sites* to include contiguous aquatic habitat where parameters of interest could be expected to mix readily. *Points* were the actual locations that we sought to resample over time. Water level fluctuations prevented us from always revisiting the same sample location; thus, we accounted for inconsistencies in repeat sampling locations post hoc by visualizing in ArcMap and assigning spatially similar samples to *points.* We had 1–6 *points* per *site*, depending on site size. *Sites* were assigned to 1 of 6 *clusters* based on their proximity, general geomorphic setting, and hydrology. Each *cluster* contained 1–3 *sites* (Table S1).

**Table 1:** Descriptions of variables and hypotheses for inclusion in temporal (base), spatial, and grazing models

Abbreviation	Variable	Justification
Temporal variables		
*Yr*	Year: 2017 or 2018.	Water availability varies among years, influencing water levels and temperatures, and affecting concentrations. 2016–2017 had roughly double the precipitation of 2017–2018.
*Day*	Ordinal date of water sample (0–365, from Jan 1).	Seasonal differences in water availability and temperature affect constituent concentrations. Day also reflects timing of cattle arrival/biological activity.
*Flow*	Flow (1) or no flow (0) was observed at the sampling point.	Flow influences constituents by increasing oxygen/mixing and flushing. Characteristics of lentic wetlands could also affect constituents (low oxygen, high organics, redox potential, etc.)
Spatial variables		
*Point*	Spatially similar sampling location within a site where water was drawn.	If grazing effects are relatively ubiquitous over a large area, local habitat may be a stronger predictor than site.
*Site*	Sampling points receiving the same water delivery.	Constituent concentrations likely differ across sites due to differing water sources and proximity to grazing.
*Cluster*	Broader than site, categorizes sites with shared hydrology and geomorphic setting.	If grazing effects are relatively homogenous over a large area, landscape setting may be a stronger predictor than site.
Grazing variables		
*MSG*	Measure of acute grazing effects; number of months since most recent grazing occurred within 250 m buffer around sampling point.	Recent cattle grazing is more likely to contribute to higher constituent concentrations than historic grazing.
*AUMrecent*	Measure of acute grazing effects; the AUM per acre of the most recently grazed pasture within the 250 m buffer, adjusted for the area overlapped by the buffer.	Recent, high-intensity cattle grazing is more likely to contribute to higher constituent concentrations than recent, low-intensity grazing.
*PropYrsGrazed*	Measure of cumulative grazing effects; total proportion of years grazed among all pastures in buffer.	A greater proportion of years grazed is more likely to contribute to higher constituent concentrations.
a) *AUMyr* b) *AUMavg*	Measure of cumulative grazing effects; average AUM per hectare across grazed years (adjusted for the area overlapped by the buffer) for the pasture with (a) the most years grazed in the buffer and (b) the sum of the average AUM per hectare for all pastures within the buffer.	High-intensity grazing is more likely to contribute to higher constituent concentrations than low-intensity grazing.

We considered multiple approaches to evaluate relationships with grazing. Limitations in our understanding of constituent transport and processes affecting rates of decay led us to simplify assumptions and use a buffer approach similar to other studies of grazing and amphibians (Knutson et al. 1999; Piha et al. 2007; Pelinson et al. 2016; Moreira et al. 2016; Boissinot et al. 2019). We generated a 250-m radius (48.5 acre) circular buffer centered on each sample location and calculated the area of grazing land (in hectares) intersecting the buffer using ArcMap. That area was used to determine a proportion of each grazing pasture that fell within each buffer. We calculated Animal Unit Months (AUM) as the number of cattle multiplied by the duration of grazing on each pasture; we divided AUM by pasture size to obtain a density-corrected grazing intensity for each pasture. AUM was then multiplied by the proportion of the grazing unit that fell within each buffer. The resulting buffer-adjusted AUM was used to derive the grazing intensity variables associated with each sample (Table 1).

#### Constituent summaries

We used robust regression on order statistics (robust ROS) to impute censored data values below the DL (i.e., for left-censored data) and derive summary statistics for ammonia, orthophosphate, turbidity, *E. coli*, and estrogenicity. Robust ROS estimates parameters from a linear regression of uncensored values on a normal probability plot (Helsel and Cohn 1988). This method is recommended for small datasets (*N* < 50) with less than 80% censoring and can be used for datasets with one or more censoring levels. Robust ROS is a semiparametric technique that assumes the uncensored data follow a parametric distribution; thus, we first transformed each constituent to achieve lognormality. However, because robust ROS uses a distributional assumption only in imputing the censored values, it is relatively insensitive to departures from a lognormal distribution (Shumway et al. 2002). For right-censored constituents of total coliforms and enterococci, we used the nonparametric Kaplan-Meier technique to estimate complete distributions. Kaplan-Meier assumes data are from the same population but does not require any distributional assumptions and is insensitive to outliers (Kaplan and Meier 1958; Hosmer et al. 2008). All calculations were performed using the *EnvStats* packages in R (R Development Core Team 2019; Millard 2013).

#### Relationships among constituent community

We used nonparametric techniques to investigate the relationships among the constituent community over time and space. We tested pairwise correlations among constituents and water temperature, pH, and specific conductance using Kendall’s *τ* implemented in the *NADA* package in R (Lee 2017). Kendall’s *τ* is derived from a nonparametric test on ranks and ranges from − 1 (mean ranks of two variables behave oppositely) to + 1 (mean ranks of two variables increase together).

We used permutational multivariate analysis of variance (perMANOVA) to determine whether there were significant differences in the distributions of water quality constituents between groups consisting of *year*, *month*, and *site*. We substituted left-censored data with a value of 0.5*DL. When FIB data were outside the linear range of the standard curve, we used the maximum count value in the analysis. We relativized all continuous environmental variables (constituents, ordinal day, pH, water temperature, and specific conductance) by maximum value to standardize across different measurement scales. We removed one outlier value of > 24,000 in enterococci. PerMANOVA was performed using the “adonis” function in R’s *vegan* package on pairwise Euclidean dissimilarity indices, and the significance of the partitioned variance was assessed using 1000 permutations (Anderson 2017; Oksanen et al. 2019).

#### Differences in constituent distributions

We used the Peto and Peto (1972) version of the Wilcoxon test to examine differences in Kaplan-Meier distributions of right-censored constituents among sites with adequate sample sizes (Big Springs N, Big Springs S, Kane Ditch, New Acquisition, Rock Island, and S Kirk) (Helsel 2012). We assessed pairwise differences in distributions among sites using Bonferroni-adjusted *p* values. A significant result indicates the probability distribution of a constituent being below a given level along a concentration gradient differs between the groups (i.e., the distribution functions differ). Cumulative distribution function (CDF) curves were used to qualitatively assess differences in constituent distributions among sites.

#### Models of effects of temporal, spatial, and grazing predictors on constituents

We used censored regression (tobit) models to examine the effects of grazing variables on each constituent independently. We excluded highly censored nitrate + nitrite and nitrite, as well as sites with small sample sizes and where grazing information was unavailable, including Peninsula Rd, which was sampled in only 2017 (*N* = 2 samples of 1 point each), Military Crossing (4 samples in 2 years), and New Acquisition (11 samples in 2 years, but insufficient grazing information). Our final dataset included *N* = 104 samples across 6 sites: Big Springs N, Big Springs S, Kane Ditch, Rock Island, Ray, and S Kirk.

We fit models for left-censored constituents of ammonia, orthophosphate, turbidity, *E. coli*, and estrogenicity using the *NADA* package in R (Lee 2017). For right-censored total coliforms and multiply censored enterococci, we used the *survival* package in R (Therneau 2015) and set the upper limits of right-censored values to infinity. Constituents, except for estrogenicity, followed a lognormal distribution. For estrogenicity, we used a Box-Cox transformation (*λ* = 0.641) to achieve approximate normality in residuals. We assessed model fit using the log-likelihood test statistic (*G*^2^_0_), likelihood-*r*^2^, and residual plots, as in Helsel (2012).

We selected models with 5 or fewer parameters based on a priori hypotheses listed in Table 1. We used a build-up model fitting approach to compare models within three hierarchical groups to understand the relative importance of spatiotemporal processes and grazing. The first group compared models of temporal processes and habitat types. These “base” models included combinations of *yr* (year), *day*, and *flow*. Because there was evidence for a peak in constituent concentrations at intermediate ordinal dates and even a sinusoidal relationship, we also considered models with a quadratic term, *I*(day^2^), and a polynomial term, *I*(day^3^). We carried the top-ranked base model (most parsimonious, top-ranked model with ∆AIC_*C*_ < 2) over to the second group, which compared effects of spatial scale. “Spatial scale” models evaluated the effects of *point*, *site*, and *cluster*. We followed a similar procedure including the favored predictors from the first two groups in comparisons of models for relationships with grazing. Models in the “grazing” group evaluated short-term effects of grazing by comparing buffer-adjusted predictors Months Since Grazing (*MSG*), grazing intensity in the most recent year a sampling point was grazed (*AUMrecent*), and their interaction (*MSG* × *AUMrecent*). Cumulative grazing effects were tested by comparing models including proportion of years since 2010 that had any grazing (*propYrsGrazed*), grazing intensity in most heavily grazed year since 2010 (*AUMyr*), average grazing intensity since 2010 (*AUMavg*), and their interactions *propYrsGrazed* × *AUMyr* and *propYrsGrazed* × *AUMavg*. For all comparisons, we ranked models using Akaike’s information criterion corrected for small samples (AIC_*C*_), relative log-likelihood, and model weights (*w*_i_). This hierarchical model fitting approach resulted in a final set of top models with ∆AIC_*C*_ < 2 for each constituent from which we could assess relative support for temporal, spatial, and grazing predictors using model rankings. All continuous variables were centered on their mean.

## Results

**Fig. 2:**
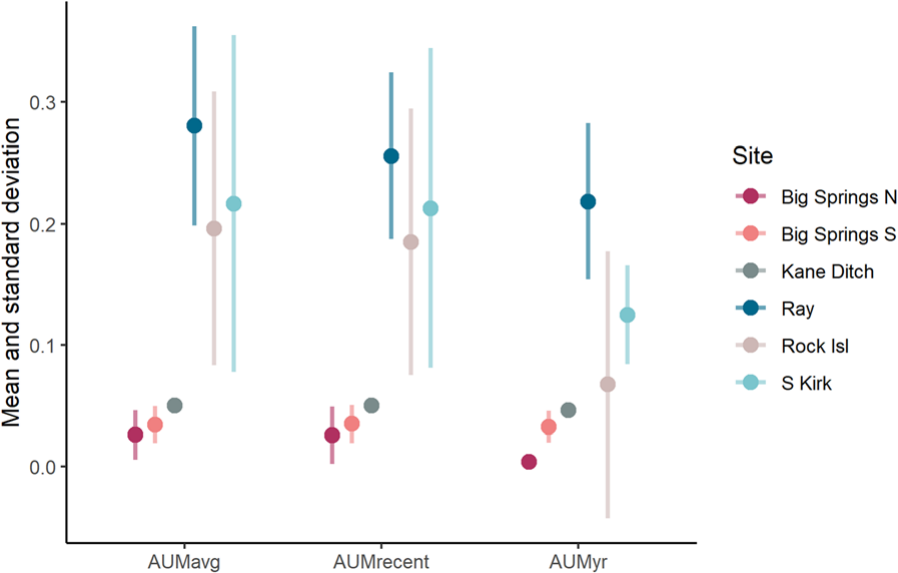
Grazing intensity variables considered in models of predictors of constituent levels, averaged by site with standard deviation. See Table 1 for information on how each variable was calculated

We collected 130 water samples across 9 sites (Fig. 1); 55 samples were collected in 2017 and 75 in 2018. Sampling locations were separated by a mean distance of 84.3 m (SD = 118.3) within sites and by a mean distance of 49.4 m (SD = 73.1) within points. We classified 70 samples across 19 points as lentic with no flow (marsh/pond) and 60 samples across 10 points as lotic with flow (stream/ditch). Sampling point buffers contained 0.37–100% (*x̅* = 82.45, SD = 36.04) grazing land, with the most recent grazing occurring between 0 and 95 (*x̅* = 41, SD = 36.6) months prior to sampling at a buffer-adjusted AUM/acre of 0.00003 to 0.295 (*x̅* = 0.063, SD = 0.075). Buffer-adjusted grazing intensity was on average highest for samples collected at Ray and lowest for samples collected at Big Springs N (Fig. 2). In general, samples in the Kane cluster were exposed to lower grazing pressures and a narrower range of grazing intensities (*AUMavg*, *AUMrecent*, and *AUMyr*) than samples in the Grazing and Rock Island clusters.

### Site and seasonal differences in surface water constituent communities

Dissolved inorganic nitrogen species including nitrate + nitrite and nitrite were low across the study area with a mean of 0.01 mg/L and 0.001 mg/L, respectively (Table 2). Because of the low concentrations and frequency of detections (datasets were > 89% censored), we did not analyze these constituents. We found significant positive relationships between turbidity and a variety of water quality constituents including ammonia, orthophosphate, total coliforms, *E. coli*, enterococci, and specific conductance (Table 3). Concentrations of *E. coli* and total coliforms were positively correlated, and *E. coli* was also positively correlated with orthophosphate and estrogenicity. Enterococci concentrations were correlated with total coliforms and *E. coli*. Results of perMANOVA indicated interannual (*R*^2^ = 0.037, *p* = 0.008, *F* = 3.89), inter-site (*R*^2^ = 0.147, *p* = 0.003, *F* = 2.02), and seasonal (*R*^2^ = 0.214, *p* = 0.001, *F* = 6.66) differences in the constituent community. Fecal indicator bacteria and estrogenicity were the only constituents showing notable inter-site differences in distribution functions and concentrations tended to be higher in sites with more years grazed at a higher stocking rate (Table S1; Fig. S1).

**Table 2:** Constituent summaries for surface water samples from Klamath Marsh National Wildlife Refuge, Oregon, 2017–2018

Constituent	*N* samples	Mean^×^	Median	Standard deviation	Percent censored^†^
Turbidity (NTRU)	121	11.1	3.60	26.6	24.0
Ammonia (mg/L as N)	121	0.04	0.01	0.12	42.2
Orthophosphate (mg/L as P)	121	0.07	0.02	0.17	0.83
Estrogenicity (ng/L)	121	0.72	0.33	0.89	52.9
Total coliforms (MPN/100 mL)	115	25,654	7300	36,981	14.8
*Escherichia coli* (MPN/100 mL)	115	313	15.0	1144	13.9
Enterococci (MPN/100 mL)	109	5833	29.0	10,184	26.6
pH	130	6.57	6.60	0.57	0.0
Specific conductance (μS/cm)	130	130	114	47.6	0.0
Temperature (°C)	117	13.5	13.8	5.24	0.0

*NTRU*, nephelometric turbidity ratio unit; *mg/L*, milligrams per liter; *N*, nitrogen; *P*, phosphorous; *MPN*, most probable number; *mL*, milliliter; *μS/cm*, microsiemens per centimeter

×Means were calculated using imputed estimates from robust regression on order statistics (robust ROS) for turbidity, ammonia, orthophosphate, estrogenicity, and *E. coli*. Data were transformed to achieve lognormality in the censored data distribution and back-transformed. Means were calculated using the nonparametric Kaplan-Meier technique for total coliforms and enterococci, with the right-censored data set to the maximum detection limit

^†^Percentage of values within the dataset that are below the minimum lab-defined detection limit and/or outside the linear range of the upper end of the standard curve (FIB values noted as greater than in Rowe et al. (2019))

**Table 3:** Nonparametric correlation coefficients (Kendall’s *τ*) for each pair of constituents. Italicized text indicates *p* ≤ 0.05

	Turbidity	Ammonia	Orthophosphate	Estrogenicity	Total coliforms	*E. coli*	Enterococci	Water temperature	pH
Turbidity	1.000								
Ammonia	*0.255*	1.000							
Orthophosphate	*0.246*	*0.232*	1.000						
Estrogenicity	0.075	− 0.034	0.082	1.000					
Total coliforms	*0.156*	0.069	0.082	0.069	1.000				
*E. coli*	*0.187*	0.097	*0.212*	*0.224*	*0.152*	1.000			
Enterococci	*0.201*	0.021	0.120	0.109	*0.130*	*0.170*	1.000		
Water temperature	0.010	0.003	− 0.063	0.058	0.173	0.034	0.161	1.000	
pH	0.127	0.112	0.070	− 0.074	0.065	− 0.025	− 0.006	*− 0.333*	1.000
Specific conductance	*0.262*	*0.279*	0.091	*− 0.138*	*0.085*	0.018	0.073	− 0.056	− 0.056

### Effects of temporal, spatial, and grazing predictors on constituents

**Fig. 3:**
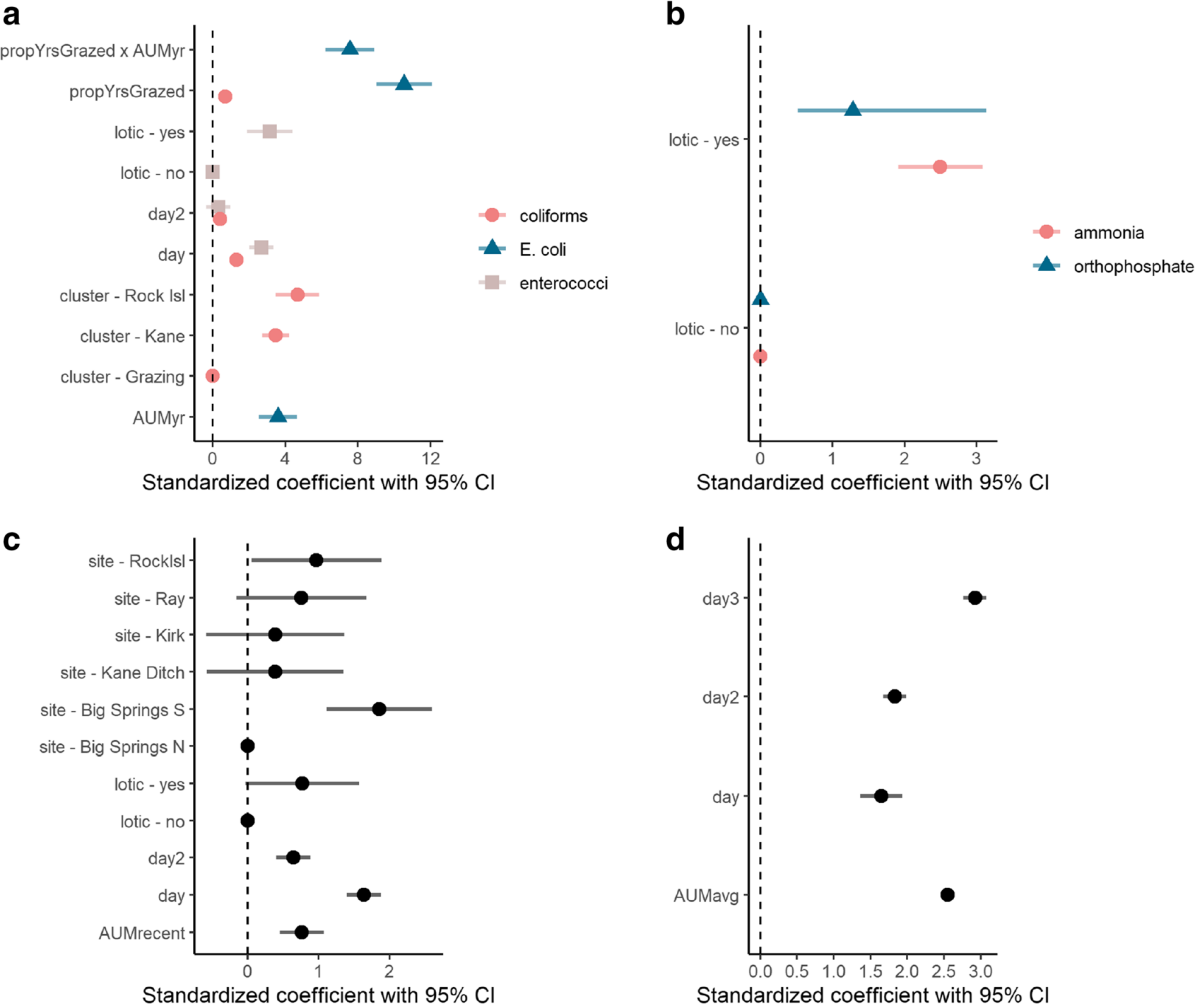
Standardized coefficients with 95% confidence intervals for **a** fecal indicator bacteria, **b** nutrients, **c** turbidity, and **d** estrogenicity. Coefficients standardized to aid in comparisons among predictors that are different scales and should be interpreted as the change in the response (constituent concentration, back transformed to original scale) with a 1 standard deviation increase in the predictor. Coefficients with confidence intervals crossing 0 are considered uninformative. Note that *point* was included in the top grazing model for orthophosphate; however, it is not shown here

**Fig. 4:**
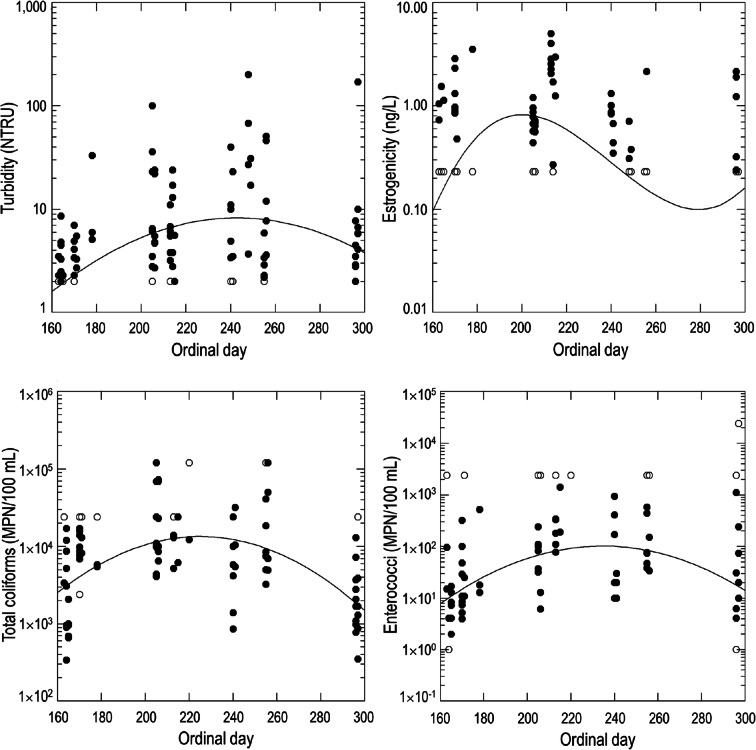
Constituent concentrations versus ordinal day across all sites. Scatterplots show uncensored (filled dots) and censored (open dots) data points with model-predicted fit lines for the best supported trend

*Day* and *flow* were consistently included in top base models for all modeled constituents (Table 4; Fig. 3). Several of the base models were improved by considering a spatial component (i.e., models including a spatial component of *site*, *point*, or *cluster* ranked higher than base models including only a temporal component; Table S2). Including a *site* predictor better explained patterns in turbidity than temporal predictors alone and variability in orthophosphate was best explained by considering *point* along with *flow*. Total coliforms varied by *day* and *cluster*, while *E. coli* levels were not well predicted by any variables in the base or spatial models. Turbidity, total coliforms, and enterococci peaked at intermediate days (between early August and early September; Fig. 4). Estrogenicity displayed a bimodal distribution, peaking in mid-July (day 200), decreasing to below detection in early October (day 280), and increasing again later in October (Fig. 4).

**Table 4:** Top grazing models (∆AIC_*C*_ **<** 2) for each water quality constituent, ranked by the Akaike information criterion corrected for small samples (AIC_*C*_). Models are described by number of parameters (*k*), AIC_*C*_, difference between given model and the model with lowest AIC_*C*_ (∆AIC_*C*_), model weight (*w*_i_), and the likelihood-*r*^2^ (*R*^2^). Bold text indicates the model we considered the top model (lowest number of parameters within ∆AIC_*C*_ of 2). See Table S2 for full grazing model set

Constituent	Grazing model	*k*	AIC_*C*_	∆AIC_*C*_	*w*_i_	*R*^2^
Orthophosphate	***Flow + point***	22	− 452.87	0.00	0.38	0.710
	Flow + point + AUMavg	23	− 451.04	1.83	0.15	0.714
Ammonia	Flow + AUMavg	2	− 176.43	0.00	0.26	0.111
	**Flow**	1	− 175.97	0.46	0.21	0.088
	Flow + AUMrecent	2	− 175.95	0.48	0.21	0.107
Turbidity	Day + *I*(day^2^) + flow + site + propYrsGrazed × AUMyr	11	606.39	0.00	0.38	0.420
	**Day +** ***I*(day**^**2**^**) + flow + site + AUMrecent**	9	607.93	1.54	0.18	0.382
Total coliforms	Day + *I*(day^2^) + cluster + propYrsGrazed × AUMavg	7	1740.90	0.00	0.44	0.396
	**Day +** ***I*(day**^**2**^**) + cluster + propYrsGrazed**	5	1742.61	1.71	0.19	0.355
*E. coli*	**PropYrsGrazed × AUMyr**	3	1002.83	0.00	0.55	0.094
Enterococci	**Day +** ***I*(day**^**2**^**) + flow**	3	866.96	0.00	0.32	0.191
	Day + *I*(day^2^) + flow + MSG	4	868.66	1.70	0.14	0.195
	Day + *I*(day^2^) + flow + propYrsGrazed	4	868.75	1.79	0.13	0.195
Estrogenicity	Day + *I*(day^2^) + *I*(day^3^) + propYrsGrazed × AUMyr	6	276.05	0.00	0.39	0.229
	**Day +** ***I*(day**^**2**^**) +** ***I*(day**^**3**^**) + AUMavg**	4	277.97	1.92	0.15	0.186

Top models for turbidity, estrogenicity, total coliforms, and *E. coli* included a grazing predictor variable. Ammonia, orthophosphate, and enterococci were relatively unaffected by grazing, but instead influenced strongly by whether a sample was collected at a lotic versus lentic point. Concentrations at lotic sites were 2.5 times higher and 3.1 times higher than at lentic sites for ammonia and enterococci, respectively. *Flow* was also included in top grazing models for turbidity, but the confidence interval crossed 0 (95% CI: − 1.07 to 0.54). Orthophosphate was spatially variable by *point*, and turbidity varied by *site* and *day*, peaking mid-season. The interaction between total proportion of years grazed among all pastures in the buffer (*propYrsGrazed*) and the most years grazed in the buffer (*AUMyr*) was associated with higher concentrations of *E. coli*, suggesting accumulating effects of grazing over time. Specifically, the slope of the effect of the average AUM per hectare across grazed years on *E. coli* increased by 67.84 for each increase in proportion of years the site was grazed. This *propYrsGrazed × AUMyr* interaction term was also influential for turbidity and estrogenicity (Table 4); however, competing top models included the grazing predictors *AUMrecent* (AUM/ha of the most recently grazed pasture within the buffer) and *AUMavg* (sum of the average AUM/ha for all pastures in the buffer), respectively. Total coliforms were slightly higher at points with higher *propYrsGrazed*; however, this effect was minimal compared to the quadratic effect of *day* and *cluster.*

## Discussion

This study was designed to provide information on the quality of surface waters at KMNWR and a baseline for monitoring factors related to grazing and *R. pretiosa* habitat in a landscape where they co-occur. We identified several water quality constituents that were related to our grazing metrics and can be easily monitored by resource managers to evaluate habitat conditions in areas with species of conservation concern. Top-ranked models for turbidity, estrogenicity, total coliforms, and *E. coli* all included a grazing predictor variable, while models for ammonia, orthophosphate, and enterococci were poorly predicted by grazing. Model-predicted effect sizes suggested a stronger relationship between *E. coli* and grazing than the collective group of total coliforms, which were better predicted by spatial and temporal variables and may be more reflective of non-fecal sources common in the environment (Fisher and Endale 1999). Without experimentally excluding other animals, we cannot explicitly link FIB levels to cattle grazing alone, as enterococci and *E. coli* occur broadly in mammals and birds. Microbial source tracking methods are needed to accurately target host-specific microorganisms and increase specificity that is currently lacking with traditional FIB methods (Byappanahalli et al. 2012; Field and Samadpour 2007). Still, the relationship between grazing and FIB found here is consistent with other studies (e.g., Canals et al. 2011; Roche et al. 2013), and points to bacteria as an informative parameter for monitoring, especially given it may be less prone to within-wetland processes such as denitrification or biotic utilization of organic nitrogen that can mask inputs and result in low concentrations of nutrients (Hopkinson 1992). Moreover, the strong correlation between turbidity and several other measured constituents provides further support for grazing effects, as turbidity has been used as an effective surrogate for grazing-related inputs of suspended sediments, phosphorous, and *E. coli* in previous studies (Mapfumo et al. 2002; Davies-Colley et al. 2008; Hughes et al. 2016). Estrogens are common in surface waters (Adeel et al. 2017), but more detailed information on the exact mixture of compounds is needed to differentiate between livestock, anthropogenic, and natural sources, which could be included in future monitoring efforts. Despite these limitations, our results suggest monitoring strategies that include measurements of turbidity, FIB, and estrogenicity could help managers detect grazing-related water quality changes and identify particularly impacted habitats in need of more detailed evaluation via microbial source tracking, specific steroidal estrogens, or other methods.

The 10-year grazing history at KMNWR allowed us to quantify differences in water quality constituents associated with long- and short-term grazing across a range of stocking densities. Because there were uncertainties regarding the influence of grazing frequency versus intensity, we compared multiple metrics in our models. The interaction between the proportion of years grazed and dominant-year buffer-adjusted AUM, as well as average buffer-adjusted AUM, was present in multiple top-ranked models, providing some support for cumulative effects. Interannual or within-season carryover effects relative to grazing duration are not commonly reported in the literature (Tiedemann et al. 1987; Myers and Whited 2012; Roche et al. 2013; Pilon et al. 2018). The grazing variables in our models have some advantages over metrics in other correlative studies that do not address density and duration, but they do not necessarily reflect how cattle congregate in the different pastures. The buffer-adjusted calculations assume a generally consistent relationship between cattle density and the receiving water that we sampled. Quantitative information regarding native ungulate use was also unavailable for model incorporation. However, observations from KMNWR staff confirm that the native ungulates (mainly elk) in the region use wetland areas less during the summer than the late fall (outside our sampling period) when cattle are typically absent.

Our results emphasize the need for consistent spatial and temporal monitoring to capture seasonal changes in water quality across a broad range of habitat types. We found *day* and *flow* to be important predictors in top models for all constituents. Cattle grazed the refuge between mid-May and mid-October depending on the pasture. We observed significant effects of date on turbidity, total coliforms, and enterococci in which levels peaked in late summer then declined into fall. Livestock use often increases turbidity in wetlands (Mapfumo et al. 2002; Knutson et al. 2004; Schmutzer et al. 2008) and in flashier systems, suspended solids can lag the time of grazing, peaking with late rains that transport solids or erode unvegetated surfaces (Gary et al. 1983; Pilon et al. 2018; Pulley and Collins 2019). Bacterial life cycles can contribute to temporal trends, and other studies in the western US have noted a late summer peak in FIB correlated with high cattle densities and warm, low-water conditions as well as in runoff after rainfall (Tiedemann et al. 1988; Roche et al. 2013). Estrogenicity had a bimodal distribution over the summer, which could be a direct effect of grazing (Kolodziej and Sedlak 2007) or could reflect peaks in sources of natural estrogens such as algae, vegetation, or other wildlife (Lambert and Edwards 2017). To our knowledge, the seasonality of estrogens in grazed habitats has not been previously reported, and more research is needed in other habitat types (e.g., riparian streams, oligotrophic lakes, temperate lowlands) and climatic regimes across a gradient of grazing pressures.

Turbidity, ammonia, orthophosphate, and enterococci were all found at higher concentrations in lotic compared to lentic habitats. A range of characteristics differentiate our lotic and lentic sampling locations and could affect rates of deposition, assimilation, flushing, and chemical processes like nitrification (Newcomer Johnson et al. 2016). Lentic sites were emergent marshes with minimal flow, abundant vegetation, shallow depth, high surface area per water volume, and water temperatures with greater maxima and diel ranges. Lotic sites primarily consisted of ditches excavated to drain water from large areas of organic marsh. Ditches have been prominent conduits on KMNWR for decades and in similar landscapes can serve as nutrient collection and delivery pathways (Skaggs et al. 1994; Schilling and Spooner 2006). Ditches and other hydrological modifications are common in marsh habitats across the range of *R. pretiosa* (USFWS 2014)*.*

These spatial and temporal trends in constituent concentrations suggest *R. pretiosa* could be exposed to elevated concentrations during early ontogenetic stages. Seasonal peaks in constituent concentrations corresponded with the period of *R. pretiosa* larval development and metamorphosis in KMNWR. Other congeneric frogs show stage-specific susceptibility to contaminants (e.g., Greulich and Pflugmacher 2003). Survivorship in early life stages can be linked to recruitment, with density dependence buffering some effects on population size (Schmutzer et al. 2008; Babini et al. 2016). Most *R. pretiosa* life stages inhabit vegetated, still-water microhabitats, so exposure to constituents in lotic sites in our study area may be limited. Specific data on habitat selection and use (e.g., radio telemetry tracking) in combination with environmental sampling could help clarify locations and timing of potential exposure (Swanson et al. 2018).

Concentrations of orthophosphate, estrogenicity, and FIB at some locations were elevated compared to established health standards and other levels reported in the region, underscoring the value of more effects-based studies on amphibians and particularly *R. pretiosa*. Five percent of our orthophosphate samples exceeded the aquatic criteria of 0.33 mg/L instituted by New York for wetlands (no nutrient criteria available for Oregon; https://www.epa.gov/nutrient-policy-data/state-progress-toward-developing-numeric-nutrient-water-quality-criteria). Elevated phosphorus concentrations can reduce amphibian development and survival (Hamer et al. 2004), but these effects vary by species (Egea-Serrano et al. 2012). Estrogenicity was observed in about 50% of the samples and about 25% of those were above the US Environmental Protection Agency (EPA) effects-based trigger value of 1 ng/L (Conley et al. 2017). Exposure to endocrine-disrupting steroid hormones in selected other amphibians even at low concentrations can alter sex ratios (Lambert et al. 2015; Lambert and Skelly 2016), affect development (Iguchi et al. 2001), and alter mating behavior (Hoffmann and Kloas 2012). No water quality standards or effects thresholds for FIB exist for wildlife, but levels of FIB at our refuge sites influenced by grazing were comparatively higher than values reported from other studies in the western US. For example, our sample-wide mean concentrations of total coliforms (25,654 MPN/100 mL) and *E. coli* (313 MPN/100 mL) greatly exceeded concentrations found across 12 US Forest Service–managed public land grazing allotments in northern California (mean total coliform concentrations of 82 MPN/100 mL and *E. coli* concentrations of 40 MPN/100 mL) (Roche et al. 2013). The highest concentration of *E. coli* (mean 496 ± 1002 MPN/100 mL) occurred at a site (Ray) where sample collection locations were directly exposed to grazing during the year of collection in 2018, and these samples had the highest mean buffer-adjusted AUM/acre out of samples from any other site (Fig. 2; Fig. S1). Currently, there are no field studies documenting the direct effects of FIB on amphibians but exposure to *E. coli* in laboratory disease challenges altered the proteins expressed in ranid skin secretions potentially reducing frog immunity (Xiao et al. 2014). Because disruptions to the amphibian skin microbiome can have implications for pathogen defenses and immunoresponses (Jani and Briggs 2014; McCoy and Peralta 2018), more information is needed on the effects of FIB and other prevalent constituents on skin defenses.

## Conclusions

Existing data on the effects of contaminants on amphibians primarily come from laboratory studies, and outcomes at laboratory-tested concentrations may not translate to natural systems where other processes interact to mask or enhance effects (Egea-Serrano et al. 2012). We collected field data to examine the spatiotemporal dynamics and potential influence of grazing on multiple water quality constituents in habitats occupied by a threatened amphibian. We employed seasonal and multiyear sampling and analyses for censored data, which is rare in water quality studies. These baseline water quality data provided a useful comparison to other managed lands and serve as a basis for future monitoring in our study site and region. Our field-measured constituent concentrations also inform ecologically relevant exposures for *R. pretiosa* and other species in laboratory-designed effects studies. Effects on individual survival or fitness may not always carry to the population level, so a combination of controlled laboratory trials and field data are needed to understand potential organismal and population-level effects at various constituent concentrations.

## Supplementary information

ESM 1 (DOCX 157 kb)

## Data Availability

All data generated for the study are available at 10.5066/P9A8OX93 (Rowe et al., 2019).
