# Supplementary material for: Monitoring wetland water quality related to livestock grazing in amphibian habitats
Source: Environ Monit Assess. 2021 Jan 13;193(2):58. doi: 10.1007/s10661-020-08838-6 (PMC7806560; doi:10.1007/s10661-020-08838-6)
Supplement: Supplementary file 1 — (DOCX 157 kb) [file 10661_2020_8838_MOESM1_ESM.docx]

SUPPLEMENTARY FIGURES AND TABLES

Monitoring wetland water quality related to livestock grazing in amphibian habitats

KL Smalling, JC Rowe, CA Pearl, LR Iwanowicz, CE Givens, CW Anderson, BM McCreary, MJ Adams, Environmental Monitoring and Assessment, 2021.


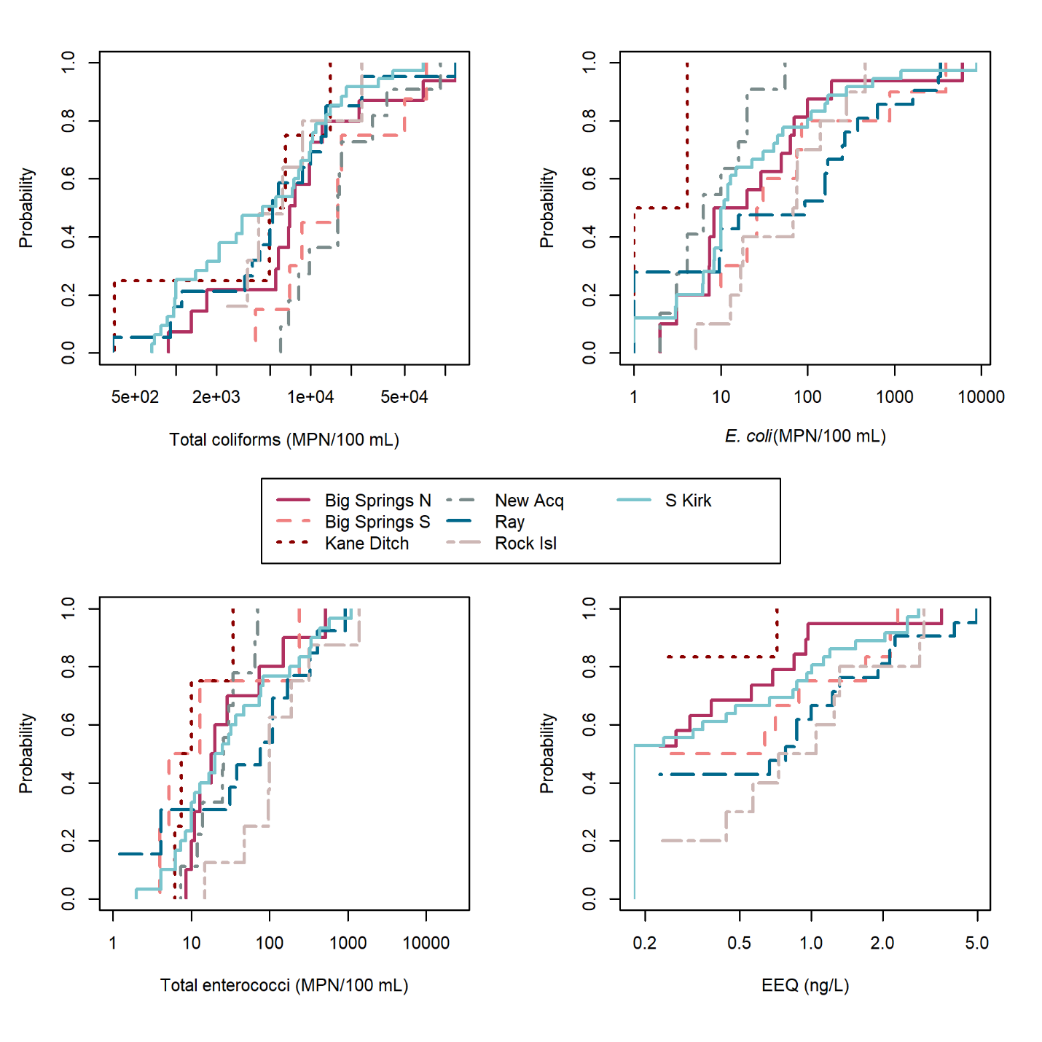


**Fig. S1**. Cumulative distribution functions (CDF) of ranked fecal indicator bacteria (FIB; all in MPN/100 mL) and estrogenicity (in ng/L) data by site. Sites are differentiated by line color and type, as shown in the legend. Each line represents the likelihood of the given constituent concentration (x-axis) occurring in the sample population for the site, by percentiles (y-axis). Sites with significantly different distributions are reported in text.

**Table S1.** Number of surface water samples collected at nine sites influenced by a range of grazing intensities between 2010 and 2018. Habitat describes the general hydrology of the site along with the percentage of samples collected at lotic sources.

| **Site** | **N** | **AUM/acres×** | **Years Grazed×** | **Cluster** | **Habitat** |
| --- | --- | --- | --- | --- | --- |
| Big Springs N | 22 | 0.00-0.74 | 2010-2018 | Kane | Stream with limited groundwater (GW) **Lotic = 36.4%** |
| Big Springs S | 16 | 0.00-0.66 | 2012-2013 | Kane | Stream with limited GW  **Lotic = 37.5%** |
| Kane Ditch | 8 | 0.00-0.66 | 2012-2013 | Kane | Ditch with appreciable GW **Lotic = 50%** |
| Rock Island | 10 | 0.00-5.09 | 2010-2018 | Rock Island | Marsh/ditch complex  **Lotic = 20%** |
| Peninsula Rd | 2 | 0.00-1.56 | 2010-2018 | Peninsula Rd | Ditch with minimal GW  **Lotic = 100%** |
| Military Crossing | 4 | - | - | Military Cr | Marsh adjacent river. **Lotic = 25%** |
| New Acquisition | 11 | - | - | New Acquisition | Marsh.  **Lotic = 0%** |
| Ray | 21 | 0.00-2.24 | 2010, 2016, 2018 | Grazing | Marsh adjacent ditch  **Lotic = 42.9%** |
| S Kirk | 36 | 0.00-2.24 | 2010, 2016, 2018 | Grazing | Marsh  **Lotic = 19.2%** |

AUM, animal unit month; × Adjacent grazing/non-grazed units falling within the 250 m buffer around samples are included in value ranges. - indicates grazing history was unavailable for the site.

**Table S2.** Final grazing models including predictors (if any) carried over from top-ranking base ‘temporal’ models and ‘spatial’ models using build-up model fitting approach. Models are described by number of parameters (*k*), AIC*_C_*, difference between given model and the model with lowest AIC*_C_* (∆AIC*_C_*), relative log-likelihood (*LL*), and model weights (*w_i_*).

| **Constituent** | **Grazing model** | ***k*** | **AIC*_C_*** | **∆AIC*_C_*** | ***LL*** | ***w_i_*** |
| --- | --- | --- | --- | --- | --- | --- |
| orthophosphate | flow + point | 22 | -452.87 | 0.00 | 1.00 | 0.38 |
|  | flow + point + AUMavg | 23 | -451.04 | 1.83 | 0.40 | 0.15 |
|  | flow + point + AUMyr | 23 | -450.73 | 2.14 | 0.34 | 0.13 |
|  | flow + point + propyrsGrazed×AUMyr | 25 | -450.11 | 2.76 | 0.25 | 0.10 |
|  | flow + point + AUMrecent | 23 | -450.00 | 2.87 | 0.24 | 0.09 |
|  | flow + point + propyrsGrazed | 23 | -449.51 | 3.36 | 0.19 | 0.07 |
|  | flow + point + MSG | 23 | -449.48 | 3.39 | 0.18 | 0.07 |
|  | flow + point + propyrsGrazed×AUMavg | 25 | -444.14 | 8.74 | 0.01 | 0.00 |
|  | flow + point + MSG×AUMrecent | 25 | -443.01 | 9.86 | 0.01 | 0.00 |
| ammonia | flow + AUMavg | 2 | -176.43 | 0.00 | 1.00 | 0.26 |
|  | flow | 1 | -175.97 | 0.46 | 0.80 | 0.21 |
|  | flow + AUMrecent | 2 | -175.95 | 0.48 | 0.79 | 0.21 |
|  | flow + AUMyr | 2 | -173.93 | 2.50 | 0.29 | 0.08 |
|  | flow + MSG | 2 | -173.89 | 2.54 | 0.28 | 0.07 |
|  | flow + propyrsGrazed | 2 | -173.87 | 2.56 | 0.28 | 0.07 |
|  | flow + propyrsGrazed×AUMavg | 4 | -173.30 | 3.13 | 0.21 | 0.05 |
|  | flow + MSG×AUMrecent | 4 | -172.05 | 4.38 | 0.11 | 0.03 |
|  | flow + propyrsGrazed×AUMyr | 4 | -170.76 | 5.67 | 0.06 | 0.02 |
| turbidity | day + *I*(day^2^) + flow + site + propyrsGrazed×AUMyr | 11 | 606.39 | 0.00 | 1.00 | 0.38 |
|  | day + *I*(day^2^) + flow + site + AUMrecent | 9 | 607.93 | 1.54 | 0.46 | 0.18 |
|  | day + *I*(day^2^) + flow + site + AUMavg | 9 | 608.47 | 2.08 | 0.35 | 0.13 |
|  | day + *I*(day^2^) + flow + site | 8 | 608.62 | 2.23 | 0.33 | 0.12 |
|  | day + *I*(day^2^) + flow + site + AUMyr | 9 | 610.21 | 3.82 | 0.15 | 0.06 |
|  | day + *I*(day^2^) + flow + site + propyrsGrazed | 9 | 610.92 | 4.53 | 0.10 | 0.04 |
|  | day + *I*(day^2^) + flow + site + MSG×AUMrecent | 11 | 611.04 | 4.65 | 0.10 | 0.04 |
|  | day + *I*(day^2^) + flow + site + MSG | 9 | 611.07 | 4.68 | 0.10 | 0.04 |
|  | day + *I*(day^2^) + flow + site + propyrsGrazed×AUMavg | 11 | 612.64 | 6.25 | 0.04 | 0.02 |
| total coliforms | day + *I*(day^2^) + cluster + propyrsGrazed×AUMavg | 7 | 1740.90 | 0.00 | 1.00 | 0.44 |
|  | day + *I*(day^2^) + cluster + propyrsGrazed | 5 | 1742.61 | 1.71 | 0.42 | 0.19 |
|  | day + *I*(day^2^) + cluster + AUMyr | 5 | 1743.35 | 2.46 | 0.29 | 0.13 |
|  | day + *I*(day^2^) + cluster | 4 | 1744.18 | 3.29 | 0.19 | 0.09 |
|  | day + *I*(day^2^) + cluster + MSG | 5 | 1745.47 | 4.57 | 0.10 | 0.05 |
|  | day + *I*(day^2^) + cluster + AUMavg | 5 | 1746.07 | 5.17 | 0.08 | 0.03 |
|  | day + *I*(day^2^) + cluster + propyrsGrazed×AUMyr | 7 | 1746.10 | 5.21 | 0.07 | 0.03 |
|  | day + *I*(day^2^) + cluster + AUMrecent | 5 | 1746.41 | 5.51 | 0.06 | 0.03 |
|  | day + *I*(day^2^) + cluster + MSG×AUMrecent | 5 | 1748.11 | 7.22 | 0.03 | 0.01 |
| *E. coli* | propyrsGrazed×AUMyr | 3 | 1002.83 | 0.00 | 1.00 | 0.55 |
|  | intercept only | 0 | 1006.18 | 3.35 | 0.19 | 0.10 |
|  | MSG | 1 | 1006.86 | 4.02 | 0.13 | 0.07 |
|  | AUMavg | 1 | 1006.96 | 4.12 | 0.13 | 0.07 |
|  | propyrsGrazed | 1 | 1007.21 | 4.37 | 0.11 | 0.06 |
|  | AUMyr | 1 | 1007.71 | 4.87 | 0.09 | 0.05 |
|  | AUMrecent | 1 | 1008.03 | 5.20 | 0.07 | 0.04 |
|  | propyrsGrazed×AUMavg | 3 | 1008.44 | 5.61 | 0.06 | 0.03 |
|  | MSG×AUMrecent | 3 | 1010.66 | 7.82 | 0.02 | 0.01 |
| enterococci | day + *I*(day^2^) + flow | 3 | 866.96 | 0.00 | 1.00 | 0.32 |
|  | day + *I*(day^2^) + flow + MSG | 4 | 868.66 | 1.70 | 0.43 | 0.14 |
|  | day + *I*(day^2^) + flow + propyrsGrazed | 4 | 868.75 | 1.79 | 0.41 | 0.13 |
|  | day + *I*(day^2^) + flow + AUMyr | 4 | 869.07 | 2.12 | 0.35 | 0.11 |
|  | day + *I*(day^2^) + flow + AUMrecent | 4 | 869.16 | 2.20 | 0.33 | 0.11 |
|  | day + *I*(day^2^) + flow + AUMavg | 4 | 869.17 | 2.21 | 0.33 | 0.11 |
|  | day + *I*(day^2^) + flow + propyrsGrazed×AUMyr | 6 | 870.91 | 3.95 | 0.14 | 0.04 |
|  | day + *I*(day^2^) + flow + MSG×AUMrecent | 6 | 871.17 | 4.22 | 0.12 | 0.04 |
|  | day + *I*(day^2^) + flow + propyrsGrazed×AUMavg | 6 | 872.93 | 5.97 | 0.05 | 0.02 |
| estrogenicity | day + *I*(day^2^) + *I*(day^3^) + propyrsGrazed×AUMyr | 6 | 276.05 | 0.00 | 1.00 | 0.39 |
|  | day + *I*(day^2^) + *I*(day^3^) + AUMavg | 4 | 277.97 | 1.92 | 0.38 | 0.15 |
|  | day + *I*(day^2^) + *I*(day^3^) | 3 | 278.64 | 2.59 | 0.27 | 0.11 |
|  | day + *I*(day^2^) + *I*(day^3^) + AUMyr | 4 | 278.76 | 2.71 | 0.26 | 0.10 |
|  | day + *I*(day^2^) + *I*(day^3^) + AUMrecent | 4 | 278.86 | 2.81 | 0.24 | 0.09 |
|  | day + *I*(day^2^) + *I*(day^3^) + MSG | 4 | 279.72 | 3.67 | 0.16 | 0.06 |
|  | day + *I*(day^2^) + *I*(day^3^) + propyrsGrazed | 4 | 280.19 | 4.14 | 0.13 | 0.05 |
|  | day + *I*(day^2^) + *I*(day^3^) + MSG×AUMrecent | 6 | 280.65 | 4.60 | 0.10 | 0.04 |
|  | day + *I*(day^2^) + *I*(day^3^) + propyrsGrazed×AUMavg | 6 | 282.35 | 6.30 | 0.04 | 0.02 |
